# SGLT2 inhibition, uric acid and inflammation in glycemic burden-related progression of cardiac autonomic neuropathy in type 2 diabetes mellitus: insights from machine learning and mediation analysis

**DOI:** 10.3389/fendo.2026.1901683

**Published:** 2026-08-05

**Authors:** Jing Jing Wang, Jie Hu, Wu Dai, Yan Liu, Pan Pan Jiang, Jun Ye, Yong Hong Cao

**Affiliations:** 1 Department of Endocrinology, Hefei Hospital Affiliated to Anhui Medical University, The Second People’s Hospital of Hefei, Hefei, Anhui, China; 2 The Fifth Clinical Medical College of Anhui Medical University, Hefei, Anhui, China; 3 Department of Neurology, The First Affiliated Hospital of Xiamen University, Xiamen, China; 4 Department of Neuroscience, The First Affiliated Hospital of Xiamen University, Xiamen, China; 5 School of Medicine, Xiamen University, Xiamen, China

**Keywords:** causal mediation analysis, diabetic cardiac autonomic neuropathy, machine learning, sodium-glucose cotransporter 2 inhibitors, uric acid

## Abstract

**Objective:**

To identify independent predictors of diabetic cardiac autonomic neuropathy (DCAN) deterioration in patients with type 2 diabetes mellitus (T2DM), develop machine learning (ML)-based risk prediction models, and explore the underlying mediation of glycemic burden.

**Methods:**

This prospective cohort study included 293 T2DM patients who underwent standardized Ewing testing at baseline and follow-up. Univariable and multivariable logistic regression were utilized to identify predictors. Restricted cubic splines (RCS) were applied to evaluate non-linear relationships. Nine ML algorithms were developed and evaluated using AUC and calibration metrics, with SHAP values illustrating feature importance. Mediation analysis was performed to investigate whether longitudinal biomarker changes accounted for the association between HbA1c and DCAN deterioration.

**Results:**

During follow-up, 80 patients (27.3%) experienced DCAN deterioration. Multivariable logistic regression identified elevated HbA1c at baseline as an independent risk factor, while SGLT2 inhibitor use was significantly associated with a lower risk of deterioration. RCS analysis revealed a continuous linear risk increase for HbA1c at baseline, whereas the platelet-to-lymphocyte ratio (PLR) at baseline exhibited an inverted U-shaped relationship. Among the ML algorithms, KNN achieved the highest AUC among models meeting the prespecified calibration criterion (AUC = 0.913; Brier score = 0.084; calibration slope = 0.701; calibration intercept = −0.053). SHAP analysis confirmed SGLT2 inhibitor use, HbA1c, and PLR as the top three predictors. Mediation analysis demonstrated that longitudinal increases in uric acid (ΔUA) and white blood cell count (ΔWBC) accounted for 20.6% and 12.9% of the association between HbA1c and DCAN progression, respectively.

**Conclusions:**

SGLT2 inhibitor use is significantly associated with a lower risk of DCAN deterioration in T2DM patients, whereas elevated HbA1c levels correlate with disease progression, potentially involving pathways of exacerbated uric acid metabolism and systemic inflammation. Furthermore, the KNN-based ML model serves as a promising proof-of-concept tool for clinical risk stratification that warrants future external validation.

## Introduction

Diabetes mellitus remains a major global public health challenge and has emerged as one of the leading causes of death and disability worldwide. In 2021, approximately 529 million people were living with diabetes globally, and this number is going to exceed 1.31 billion by 2050 (1, 2). Notably, China has the largest population of individuals with diabetes, which is estimated to surpass 174 million by 2045 (3). Chronic hyperglycemia can induce a myriad of multisystemic chronic complications affecting various target organs. These complications not only significantly deteriorate patients’ quality of life but also impose a profound disease burden on individuals, families, and healthcare systems (4). Among the diverse chronic complications of type 2 diabetes mellitus (T2DM), cardiovascular disease represents the leading cause of morbidity and mortality. More than half of all diabetes-related deaths worldwide are attributable to cardiovascular events, with this proportion reaching over 60% in high-income countries (5, 6).

Within the framework of preventing and managing cardiovascular complications in T2DM, diabetic cardiac autonomic neuropathy (DCAN) remains a critical yet long-underestimated clinical target. DCAN is a specific complication characterized by the damage to cardiac sympathetic and parasympathetic nerve fibers and the imbalance of neuromodulatory function. It is often asymptomatic in its early stages, leading to a high rate of missed diagnoses and clinical neglect. In advanced stages, it can manifest as resting tachycardia, orthostatic hypotension, syncope, silent myocardial infarction, cardiac arrest, and even sudden cardiac death (7–9). Current evidence-based medicine has established that DCAN is not only an independent risk factor for malignant arrhythmias, acute myocardial infarction, and sudden cardiac death in patients with T2DM, but also exacerbates the risk of asymptomatic hypoglycemia and accelerates the progression of heart failure and diabetic nephropathy. Aside from long-term intensive glycemic control, there is currently no universally recognized specific treatment capable of effectively delaying or reversing its progression. Traditional neurotrophic and antioxidant agents, such as mecobalamin and α-lipoic acid, offer only short-term symptomatic relief. They lack high-quality evidence demonstrating efficacy in halting or reversing nerve damage and reducing hard cardiovascular endpoints. Consequently, there is an urgent need to explore novel therapeutic strategies that simultaneously provide glucose-lowering efficacy and target organ protection to improve the prognosis of DCAN (9–11).

In recent years, the treatment philosophy for T2DM has undergone a fundamental paradigm shift from a “glycemic-control-centric” approach to a “multi-target comprehensive management model aimed at improving long-term prognosis and reducing the risk of target organ damage.” The advent and clinical application of sodium-glucose cotransporter 2 inhibitors (SGLT2i) represent a core milestone in this conceptual transformation. By selectively inhibiting the sodium-glucose cotransporter 2 in the S1 segment of the renal proximal tubule, SGLT2i reduce the renal reabsorption of glucose and sodium, thereby exerting a hypoglycemic effect through increased urinary excretion of glucose and sodium. A series of international, multicenter, large-scale, randomized, double-blind, placebo-controlled cardiovascular outcome trials (CVOTs) have consistently demonstrated that SGLT2i can significantly reduce the risks of major adverse cardiovascular events, hospitalization for heart failure, end-stage renal disease, and all-cause mortality in T2DM patients with concomitant atherosclerotic cardiovascular disease, heart failure, or chronic kidney disease. Consequently, the 2024 consensus report on T2DM management by the American Diabetes Association (ADA) and the European Association for the Study of Diabetes (EASD), as well as the Chinese Guidelines for the Prevention and Treatment of Type 2 Diabetes (2024 Edition), have uniformly recommended SGLT2i as the first-line treatment of choice for T2DM patients with high cardiovascular or renal risk factors, heart failure, or chronic kidney disease, regardless of their baseline glycated hemoglobin levels (12–15).

With advancing research into the organ-protective mechanisms of SGLT2i, their regulatory effect on cardiac autonomic function has gradually emerged as a research hot spot in both endocrinology and cardiology. Current basic research indicates that SGLT2i can alleviate autonomic nerve fiber damage under diabetic conditions and restore sympathovagal balance through multiple pathways. Several small-sample clinical studies have also found that SGLT2i can significantly improve heart rate variability (HRV)-related indices, lower resting heart rate, and delay the disease progression of DCAN in patients with T2DM (16, 17). However, other relevant clinical studies have reported that, compared to non-users, patients treated with SGLT2i ultimately showed no statistically significant differences in parameters such as HRV (18, 19). Therefore, current clinical research regarding the impact of SGLT2i on DCAN remains constrained by discrepancies in trial outcomes and inconsistent evaluation metrics.

Based on this situation, this prospective cohort study aims to investigate the impact of SGLT2i on DCAN in patients with T2DM. Furthermore, we seek to identify potential therapeutic targets to further optimize comprehensive management strategies for chronic complications in this patient population.

## Materials and methods

### Patient selection and data

This single-center prospective cohort study finally enrolled 293 patients with T2DM who underwent standardized Ewing testing at baseline and follow-up visits at Hefei Hospital Affiliated to Anhui Medical University between December 2020 and March 2026.

Inclusion criteria comprised:

1. aged ≥ 18 and < 80 years;
2. diagnosed with T2DM according to the 1999 World Health Organization (WHO) criteria;
3. maintained on a stable glucose-lowering regimen for at least 3 months prior to admission;
4. successfully completed continuous glucose monitoring (CGM) and Ewing’s tests with complete data.

Exclusion criteria comprised:

1. pregnant or lactating women;
2. patients with severe cardiovascular, cerebrovascular, hepatic, or renal diseases;
3. patients with mental or physical disabilities rendering them unable to cooperate with medical examinations;
4. patients who experienced acute diabetic complications (e.g., diabetic ketoacidosis [DKA], hyperosmolar coma, or lactic acidosis) within the previous 6 months;
5. patients with type 1 diabetes mellitus (T1DM) or other specific types of diabetes (e.g., Cushing’s syndrome);
6. patients with contraindications to Ewing’s tests, such as severe hypertension or proliferative diabetic retinopathy;
7. patients with a suspected or confirmed history of alcohol or substance abuse;
8. patients using medications affecting heart rate (e.g., β-blockers, β-agonists, epinephrine, dopamine) within 48 hours prior to the tests;
9. patients with thyroid dysfunction;
10. patients with abnormal serum potassium levels; and
11. patients with incomplete clinical or CGM data, or those lost to follow-up.

**Figure 1:**
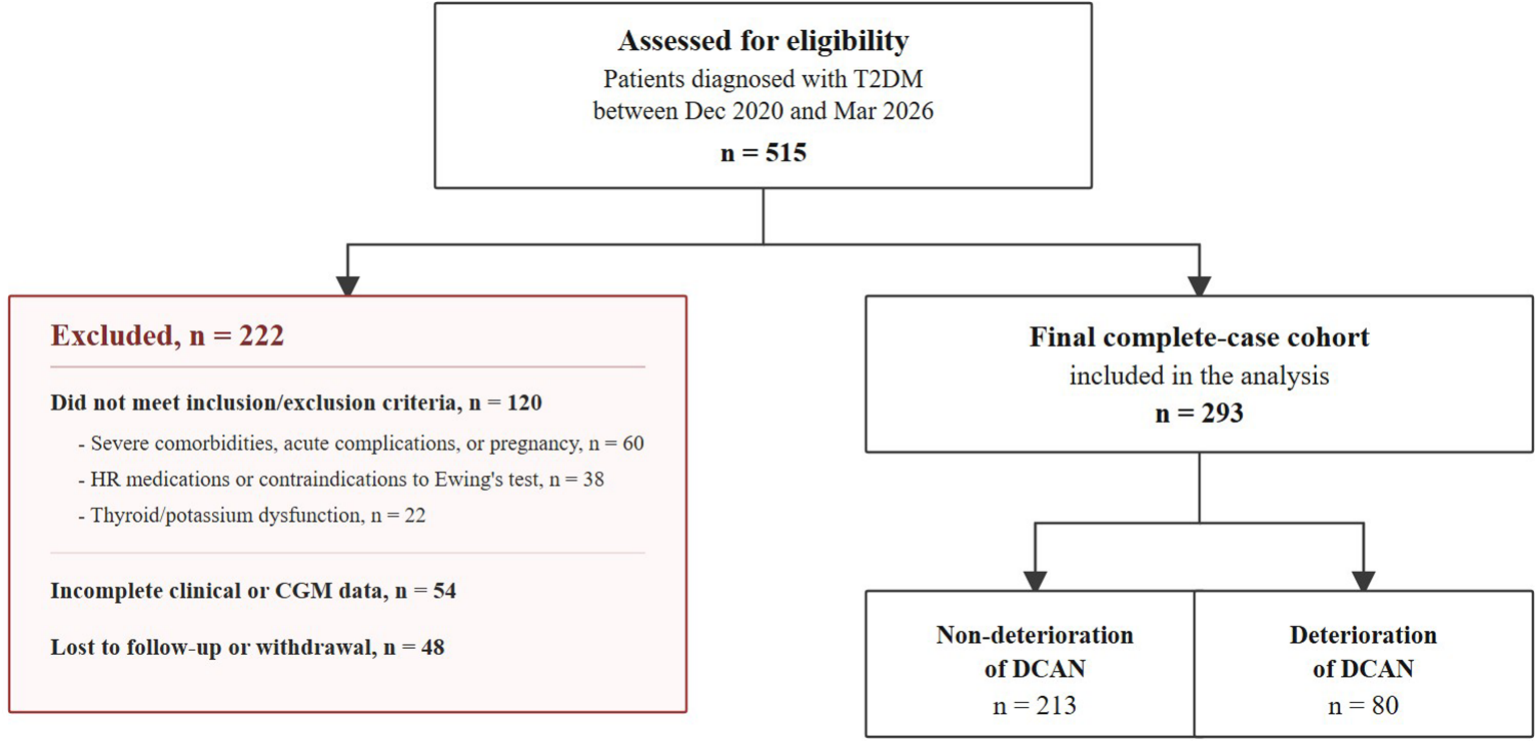
Flowchart of study population selection.

By March 2026, a total of 515 patients diagnosed with T2DM were initially screened. Among them, 222 patients were excluded due to ineligibility, loss to follow-up, withdrawal, or incomplete clinical data (**Figure 1**). Consequently, a final complete-case cohort of 293 participants (176 males and 117 females) who successfully completed the study was included in the final analysis. All variables evaluated in this final cohort had 100% complete data; therefore, no missing data imputation was required.

The study was approved by the Ethics Committee of Hefei Hospital Affiliated to Anhui Medical University, with the ethical approval number 2019-SR-084. Written informed consent was obtained from all participants included.

### Definition of outcome

The severity of DCAN was quantified using the standardized Ewing test at baseline and follow-up. The diagnosis of CAN was established based on the Ewing test, which consists of the following components: (1) Resting heart rate: A resting heart rate > 100 beats/min was considered abnormal (excluding cases of arrhythmia and cardiac dysfunction); (2) Handgrip test: Blood pressure was measured immediately after 3 minutes of sustained, forceful handgrip. An increase in systolic blood pressure (SBP) ≥ 16 mmHg (1 mmHg = 0.133 kPa) was defined as normal, whereas an increase ≤ 10 mmHg was defined as abnormal; (3) Postural blood pressure change: Blood pressure was measured in a resting supine position, followed immediately by standing. Blood pressure was then measured again within 1 minute. A decrease in SBP > 20 mmHg or a decrease in diastolic blood pressure (DBP) > 10 mmHg was considered abnormal; (4) Postural heart rate change: An increase in heart rate > 10 beats/min upon standing was considered normal, whereas an increase ≤ 10 beats/min was considered abnormal. Participants were required to rest for at least 2 minutes between each component of the Ewing test. An abnormal result in components (1), (2), and (4) was assigned 1 point each, whereas an abnormal result in component (3) was assigned 2 points. CAN was diagnosed if two or more components were abnormal, yielding a total score of ≥ 2 points (20). DCAN deterioration was defined as Δ Score = Score(follow up) - Score(baseline) > 0, whereas patients with Δ Score ≤ 0 were assigned to the non-deteriorated group. Clinically, the Ewing score represents a stage-based composite metric rather than a simple continuous variable. A confirmed increase in the score signifies the pathological impairment of additional cardiovascular reflexes, objectively reflecting a transition across CAN stages (typically from isolated parasympathetic impairment to combined sympathetic involvement). This longitudinal definition is strongly supported by classical consensus criteria and recently reaffirmed clinical frameworks (7, 9).

### Composite indices

Eight composite indices were calculated from baseline anthropometric and laboratory parameters: (i) Atherogenic Index of Plasma (AIP) = log_10_(TG/HDL-C) (21); (ii) Platelet-to-Lymphocyte Ratio (PLR) = platelet count/lymphocyte count (22); (iii) Systemic Immune-Inflammation Index (SII) = platelet × neutrophil/lymphocyte (23); (iv) Neutrophil-to-Lymphocyte Ratio (NLR) = neutrophil count/lymphocyte count (22); (v) Fibrosis-4 Index (FIB4) = (age × AST)/(platelet × √ALT); (vi) estimated Body Fat Percentage (eBFP) = 1.20 × BMI + 0.23 × age − 10.8 × sex − 5.4 (24); (vii) Non-HDL-C to HDL-C Ratio (NonHDL_HDL) = (TC − HDL-C)/HDL-C; (viii) BARD score = weighted sum of BMI ≥ 28 (1 point), AST/ALT ratio ≥ 0.8 (2 points), and presence of diabetes (1 point) (25).

### Statistical analysis

All statistical analyses were performed using R software (version 4.5.3) and Python software (version 3.14).

Continuous variables were evaluated for normal distribution using the Shapiro-Wilk test. Normally distributed continuous variables were expressed as mean ± standard deviation (SD) and compared using the Student’s t-test, whereas skewed variables were presented as median and interquartile range (IQR) and compared using the Mann-Whitney U test. Categorical variables were summarized as counts (percentages) and compared using the Pearson’s Chi-square test or Fisher’s exact test, as appropriate. Univariable and multivariable logistic regression models were sequentially performed to identify independent predictors of DCAN deterioration. To prevent model overfitting and maintain model stability in accordance with the classical events per variable (EPV ≥ 10) rule of thumb, only variables demonstrating a P-value < 0.05 in the univariable analysis were incorporated into the multivariable logistic regression model. However, established clinical confounders were subsequently force-included in the downstream models irrespective of their univariable significance.

Restricted cubic spline (RCS) analysis with four knots (5th, 35th, 65th, and 95th percentiles) was performed for HbA1c and PLR to assess potential non-linear associations with DCAN deterioration.

All Machine Learning analyses were performed using Python 3.14 with scikit-learn, XGBoost, and SHAP libraries. Ten variables comprising eight composite indices (AIP, PLR, SII, FIB4, eBFP, NonHDL_HDL, NLR, BARD) plus baseline HbA1c and SGLT2 inhibitor use served as predictor variables, and DCAN deterioration served as the binary outcome. All variables had complete data; no imputation was required. To address class imbalance (27.3% deterioration rate), class weighting (class_weight=‘balanced’) was applied during model training rather than synthetic oversampling methods such as SMOTEENN, given the modest sample size and evidence that synthetic sampling may degrade probability calibration in small datasets. Continuous features were standardized using z-score normalization for distance-based models (KNN, SVM, MLP, NB, LR); for tree-based models (RF, GBM, XGBoost, AdaBoost), raw values were used given their invariance to monotonic transformations.

The dataset was randomly divided into a training set (70%, n = 205) and a held-out test set (30%, n = 88) using stratified sampling to preserve the outcome proportion. The test set was strictly sequestered during model development and used only for final performance evaluation. Nine supervised learning algorithms spanning five families were evaluated: (i) tree-based ensembles—Random Forest (RF), Gradient Boosting Machine (GBM), XGBoost, and AdaBoost; (ii) instance-based—K-Nearest Neighbors (KNN, k = 7, distance-weighted); (iii) kernel-based—Support Vector Machine (SVM, RBF kernel); (iv) probabilistic—Naive Bayes (NB); and (v) linear/neural—Logistic Regression (LR) and Multi-Layer Perceptron (MLP, single hidden layer of 16 units with L2 regularization α = 0.1). Hyperparameter tuning was performed via 5-fold cross-validation on the training set. All tree-based models incorporated explicit regularization constraints: RF (maximum depth = 10, minimum samples per leaf = 5); GBM (maximum depth = 4, minimum samples per leaf = 8, subsample = 0.8); XGBoost (L1 regularization α = 0.5, L2 regularization λ = 1.0, minimum child weight = 5, column subsample = 0.8). Model performance was assessed on the test set using AUC, accuracy, sensitivity, specificity, and macro-averaged and weighted-averaged precision, recall, and F1 score.

To evaluate the reliability of predicted probabilities, three complementary calibration metrics were employed. First, the Hosmer-Lemeshow (H-L) goodness-of-fit test was performed: predicted probabilities were partitioned into deciles, and observed versus expected event frequencies were compared using a chi-square statistic; P > 0.05 indicated acceptable calibration. Second, calibration curves were constructed by plotting observed event fractions against predicted probabilities in ten equally-spaced bins, with calibration slope (ideal: 0.9–1.1) and intercept (ideal: −0.05 to +0.05) derived from linear regression. Third, the Brier score was computed as a composite measure of overall prediction accuracy (0 = perfect calibration, 0.25 = no discrimination), the final predictive model was selected as the one achieving the highest test-set AUC among models satisfying the H-L calibration criterion (P > 0.05). This dual criterion ensures both strong discriminative ability and reliable probability estimates.

Model optimism was quantified using the Efron-Gong bootstrap procedure with 200 resamples. For each iteration, apparent AUC was computed on the bootstrap training sample and tested on out-of-bag (OOB) observations—those not selected during resampling (~36.8% of original samples, providing genuinely unseen validation). Optimism was defined as the mean difference between apparent and OOB AUC, and optimism-corrected AUC was reported with 95% bootstrap percentile confidence intervals. The DeLong asymptotic test was used to compare AUCs among models. Decision curve analysis (DCA) was performed to evaluate net clinical benefit across threshold probability ranges.

To investigate whether longitudinal biomarker changes mediate the established relationship between baseline glycemic control and DCAN deterioration, causal mediation analysis was performed within the VanderWeele-Vansteelandt counterfactual framework, the mediation analyses were exploratory and were used to statistically decompose the observed exposure-outcome association into direct and indirect components. For each mediator, three nested models with incrementally comprehensive covariate adjustment were specified. Model 1 (Unadjusted): no covariates. Model 2 (Demographically Adjusted): adjusted for age, T2DM duration, alcohol consumption, and follow-up duration. Model 3 (Fully Adjusted): Model 2 covariates plus hypertension, SGLT2 inhibitor use, baseline Ewing score, and the baseline value of the mediator. Inclusion of the baseline mediator value is critical to isolate the “pure change” attributable to glycemic exposure from regression-to-the-mean effects. All continuous variables were standardized (z-score) prior to model fitting.

## Results

### Baseline characteristics of the study population

A total of 293 patients were included in this study, with 80 patients (27.3%) progressing to the deterioration outcome and 213 patients (72.7%) remaining stable. The median follow-up duration was 20.000 months. Compared to patients remaining stable, DCAN deterioration group exhibited significantly higher baseline levels of HbA1c (8.750 vs. 8.000, P = 0.004), WBC (6.495 vs. 5.830, P = 0.040), and ALC (1.865 vs. 1.750, P = 0.046). Moreover, the proportion of patients with a history of alcohol drinking was significantly higher in this group (38.75% vs. 24.88%, P = 0.019). Besides, the prevalence of hypertension (43.75% vs. 59.62%, P = 0.015) and the prescription rate of SGLT2 inhibitors (35.00% vs. 53.05%, P = 0.006) were significantly lower in the deterioration group. Additionally, baseline Ewing score was significantly lower in DCAN deterioration group than in control group (1.000 vs. 2.000, P < 0.001). Other demographic and clinical variables, including age, gender, BMI, and T2DM duration, showed no significant differences between the two groups (all P > 0.05)(**Table 1**).

**Table 1:** Baseline demographic and clinical characteristics of the study population.

Variables	Total (n=293)	Non-deterioration of DCAN(n=213)	Deterioration of DCAN(n=80)	*t, Z or χ²*	P
Demographic characteristics					
AGE(years;M, IQR)	59.000 (50.000, 67.000)	59.000 (51.000, 67.000)	58.500 (48.750, 63.250)	Z=-1.699	0.089
T2DM duration(months;M, IQR)	111.000 (47.000, 171.000)	108.000 (50.000, 166.000)	130.500 (47.000, 185.500)	Z=-1.157	0.247
Follow-up duration(months;M, IQR)	20.000 (12.000, 31.000)	20.000 (11.000, 32.000)	20.500 (12.000, 29.000)	Z=-0.514	0.607
BMI(kg/m2;M, IQR)	25.000 (23.389, 27.282)	25.195 (23.389, 27.160)	24.802 (23.374, 27.509)	Z=-0.785	0.432
Male(n, %)	176 (60.068)	124 (58.216)	52 (65.000)	χ²=1.116	0.291
Comorbidities and past medical history(n, %)					
CAD	16 (5.461)	13 (6.103)	3 (3.750)	χ²=0.251	0.616
DKD	56 (19.113)	38 (17.840)	18 (22.500)	χ²=0.817	0.366
DR	73 (24.915)	53 (24.883)	20 (25.000)	χ²=0.000	0.983
Hypertension	162 (55.290)	127 (59.624)	35 (43.750)	χ²=5.929	0.015
Stroke	170 (58.020)	128 (60.094)	42 (52.500)	χ²=1.377	0.241
Smoking	115 (39.249)	78 (36.620)	37 (46.250)	χ²=2.262	0.133
Drinking	84 (28.669)	53 (24.883)	31 (38.750)	χ²=5.469	0.019
Clinical characteristics					
SBP(mmHga;M, IQR)	134.000 (122.000, 146.000)	136.000 (123.000, 146.000)	134.000 (120.000, 140.500)	Z=-1.336	0.181
DBP(mmHga;M, IQR)	82.000 (74.000, 90.000)	82.000 (75.000, 88.000)	82.000 (74.000, 90.000)	Z=-0.198	0.843
HR(bpm, `x ± s)	85.338 ± 11.013	84.587 ± 11.177	87.338 ± 10.369	t=-1.913	0.057
Antidiabetic and concomitant medications(n, %)					
SGLT2I	141 (48.123)	113 (53.052)	28 (35.000)	χ²=7.591	0.006
Baseline laboratory parameters					
WBC(×109/L;M, IQR)	6.070 (5.120, 6.880)	5.830 (5.090, 6.780)	6.495 (5.203, 7.020)	Z=-2.052	0.040
RBC(×109/L, `x ± s)	4.503 ± 0.469	4.496 ± 0.455	4.522 ± 0.507	t=-0.421	0.674
Hb(g/L, `x ± s)	135.003 ± 16.014	134.233 ± 15.107	137.053 ± 18.154	t=-1.345	0.180
PLT(×109/L;M, IQR)	191.000 (152.000, 234.000)	189.000 (152.000, 237.000)	203.000 (152.000, 232.500)	Z=-0.535	0.593
ANC(×109/L;M, IQR)	3.600 (2.950, 4.280)	3.490 (2.880, 4.260)	3.700 (3.130, 4.473)	Z=-1.501	0.133
ALC(×109/L;M, IQR)	1.800 (1.470, 2.200)	1.750 (1.440, 2.160)	1.865 (1.530, 2.353)	Z=-1.996	0.046
ALT(U/L;M, IQR)	18.000 (14.000, 26.700)	18.000 (14.000, 26.000)	17.000 (12.000, 32.000)	Z=-0.670	0.503
AST(U/L;M, IQR)	19.000 (15.000, 23.000)	19.000 (16.000, 22.000)	18.500 (14.000, 26.000)	Z=-0.690	0.490
TG(mmol/L;M, IQR)	1.600 (1.010, 2.360)	1.550 (0.970, 2.240)	1.800 (1.240, 2.438)	Z=-1.869	0.062
TC(mmol/L;M, IQR)	4.240 (3.280, 5.050)	4.240 (3.150, 4.970)	4.250 (3.370, 5.270)	Z=-1.104	0.269
HDL-C(mmol/L, `x ± s)	1.120 (0.930, 1.290)	1.120 (0.970, 1.280)	1.045 (0.867, 1.290)	Z=-1.616	0.106
LDL-C(mmol/L;M, IQR)	2.550 (1.800, 3.400)	2.540 (1.760, 3.300)	2.570 (1.945, 3.590)	Z=-1.272	0.203
HbA1c(%;M, IQR)	8.300 (7.000, 9.600)	8.000 (6.900, 9.100)	8.750 (7.600, 10.200)	Z=-2.897	0.004
FCP(ng/ml, `x ± s)	1.690 (1.210, 2.560)	1.700 (1.190, 2.610)	1.610 (1.240, 2.253)	Z=-1.215	0.224
ACR(mg/g.cr;M, IQR)	15.100 (8.900, 35.800)	13.900 (8.200, 33.100)	16.450 (9.300, 38.300)	Z=-0.289	0.772
Scr(μmol/L;M, IQR)	62.700 (51.900, 76.000)	62.800 (51.100, 76.100)	62.700 (56.850, 72.800)	Z=-0.691	0.490
eGFR(mL/min/1.73m²;M, IQR)	102.457 (94.185, 111.550)	102.128 (94.185, 111.550)	102.654 (94.292, 112.659)	Z=-0.488	0.625
UA(μmol/L;M, IQR)	308.600 (259.500, 375.200)	303.700 (267.800, 370.800)	341.800 (259.100, 399.750)	Z=-1.519	0.129
CGM					
SD(M, IQR)	1.880 (1.580, 2.450)	1.830 (1.580, 2.370)	2.000 (1.600, 2.600)	Z=-1.139	0.255
MAGE(mmol/L;M, IQR)	3.800 (2.930, 5.010)	3.800 (2.960, 4.900)	4.090 (2.820, 5.768)	Z=-0.655	0.512
MG(mmol/L;M, IQR)	8.450 (7.430, 9.200)	8.350 (7.430, 9.200)	8.615 (7.522, 9.200)	Z=-1.089	0.276
TIR(%;M, IQR)	78.970 (65.000, 89.310)	78.920 (65.000, 89.310)	79.425 (62.880, 89.380)	Z=-0.006	0.995
LAGA(mmol/L;M, IQR)	9.600 (7.570, 12.200)	9.500 (7.570, 11.950)	9.985 (7.690, 13.207)	Z=-1.015	0.310
CV(%;M, IQR)	22.550 (18.830, 27.640)	23.200 (18.910, 27.460)	21.175 (18.830, 28.043)	Z=-0.623	0.533
Score(M, IQR)	2.000 (1.000, 2.000)	2.000 (2.000, 2.000)	1.000 (1.000, 2.000)	Z=-8.278	<0.001

Patients with a change in Ewing test score (follow-up score minus baseline score) > 0 were assigned to the diabetic cardiac autonomic neuropathy (DCAN) progression group (CAN+), and those with a change ≤ 0 were assigned to the non-progression group (non-CAN+). Normally distributed continuous variables were presented as mean ± standard deviation (x ± s), and compared between groups using the independent samples t-test. Non-normally distributed continuous variables were presented as median (interquartile range, IQR; M (P25, P75), where P25 and P75 indicate the 25th and 75th percentiles), and compared between groups using the Mann-Whitney U test, with the Z statistic reported. Categorical variables were presented as number (percentage, n (%)), and compared between groups using the Pearson χ² test.

ACR, urinary albumin-to-creatinine ratio; AGE, age; ALC, absolute lymphocyte count; ALT, alanine aminotransferase; ANC, absolute neutrophil count; AST, aspartate aminotransferase; BMI, body mass index; CAD, coronary artery disease; CGM, continuous glucose monitoring; CV, coefficient of variation; DBP, diastolic blood pressure; DKD, diabetic kidney disease; DR, diabetic retinopathy; eGFR, estimated glomerular filtration rate; FCP, fasting C-peptide;Hb, hemoglobin; HbA1c, glycated hemoglobin A1c; HDL-C, high-density lipoprotein cholesterol; HR, heart rate; IQR, interquartile range; LAGA, low glucose area under the curve; LDL-C, low-density lipoprotein cholesterol; M, median; MAGE, mean amplitude of glycemic excursions; MG, mean glucose; PLT, platelet; RBC, red blood cell; SBP, systolic blood pressure; Score, cardiac autonomic neuropathy score; Scr, serum creatinine; SD, standard deviation; SGLT2I, sodium–glucose cotransporter 2 inhibitor; TC, total cholesterol; TG, triglyceride; TIR, time in range; T2DM, type 2 diabetes mellitus; UA, serum uric acid; WBC, white blood cell. Unit conversion: 1 mmHg = 0.133 kPa.

### Univariable and multivariable logistic regression

To identify independent predictors for the outcome, univariable and multivariable logistic regression analyses were performed. In the univariable analysis, HbA1c, baseline Ewing score, SGLT2 inhibitor use, hypertension history, drinking history, and ALC were significantly associated with the outcome. After multivariable adjustment, higher baseline HbA1c (OR = 1.206, 95% CI 1.027-1.417; P = 0.022) and alcohol use (OR = 3.466, 95% CI 1.700-7.068; P<0.001) remained associated with greater odds of the score-increase outcome. Baseline SGLT2 inhibitor use was associated with lower odds (OR = 0.493, 95% CI 0.249-0.976; P = 0.042). Hypertension history, WBC and ALC at baseline lost their statistical significance in the fully adjusted multivariable model(**Table 2**).

**Table 2:** Multivariable logistic regression analyses for predictors of DCAN deterioration.

Variables	β	S.E	Z	P	OR (95%CI)
HbA1c	0.188	0.082	2.286	0.022	1.206 (1.027 ~ 1.417)
Score1	-1.986	0.291	-6.821	<0.001	0.137 (0.078 ~ 0.243)
SGLT2					
No					1.000 (Reference)
Yes	-0.708	0.349	-2.029	0.042	0.493 (0.249 ~ 0.976)
Hypertension					
No					1.000 (Reference)
Yes	-0.218	0.340	-0.643	0.520	0.804 (0.413 ~ 1.565)
Drinking history					
No					1.000 (Reference)
Yes	1.243	0.363	3.420	<0.001	3.466 (1.700 ~ 7.068)
WBC at baseline	0.043	0.135	0.318	0.750	1.044 (0.801 ~ 1.361)
ALC at baseline	0.026	0.366	0.071	0.943	1.026 (0.501 ~ 2.101)

### Dose-response relationship analyzed by restricted cubic splines

**Figure 2:**
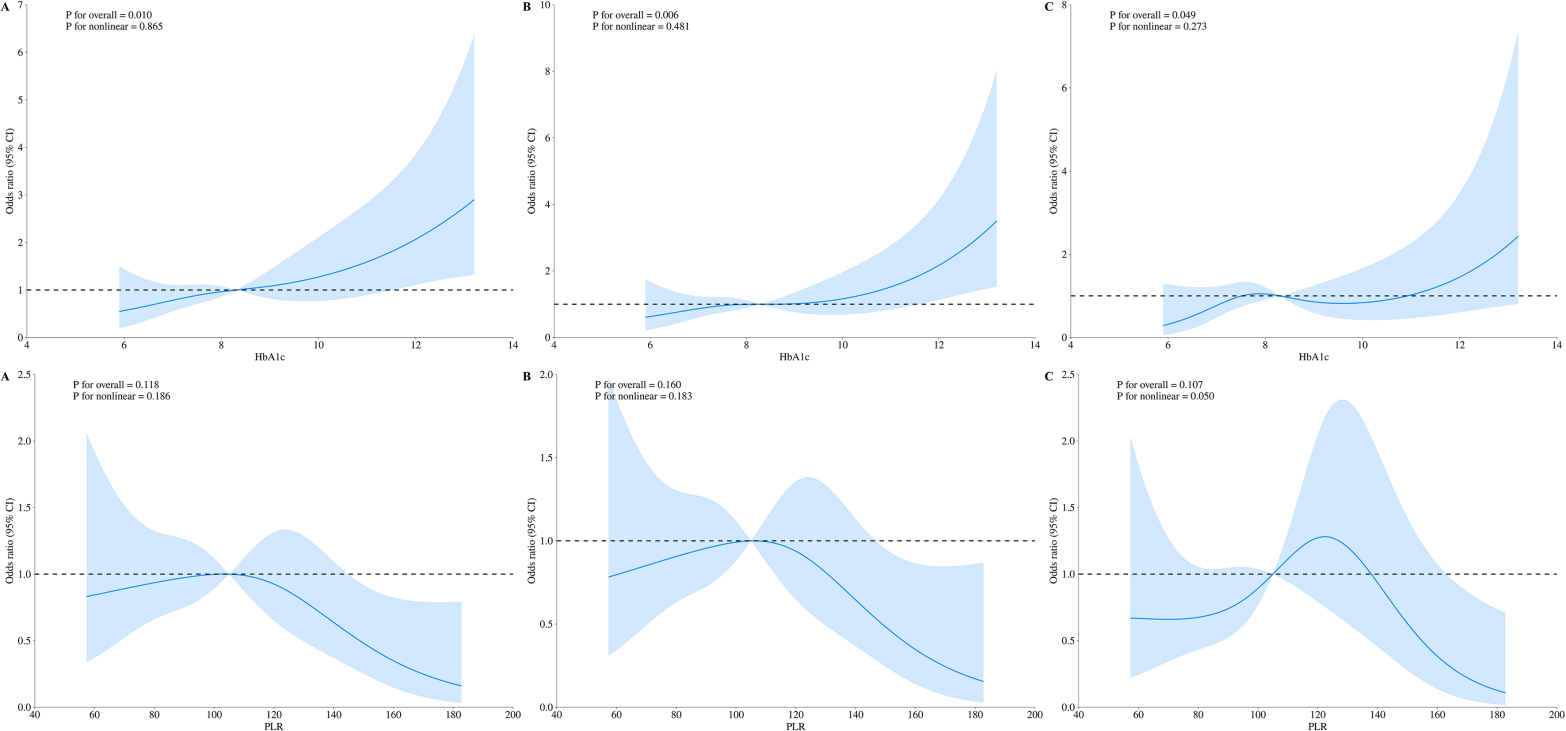
Restricted cubic spline (RCS) curves illustrating the dose-response relationship between HbA1c, PLR, and the risk of DCAN deterioration. **(A–C)** represent the crude (adjusted for nothing) (model 1), partially adjusted (adjusted for age, T2DM duration, drinking history and follow up time) (model 2), and fully adjusted models (adjusted for age, T2DM duration, drinking history, follow up time, hypertension history,SGLT2I use and Baseline Ewing score) (model 3), respectively.

For HbA1c, the RCS curves demonstrated a continuous, monotonic linear increase in the risk of DCAN deterioration. The linear association remained robust even in the fully adjusted Model 3 (P for overall = 0.049; P for nonlinear = 0.273), indicating a cumulative detrimental effect of glycemic burden without a safe plateau. While for PLR, the RCS revealed an inverted U-shaped non-linear relationship, after full multivariable adjustment (Model 3), the test for non-linearity reached borderline significance (P for nonlinear = 0.050). However, the overall association for PLR did not reach statistical significance in the fully adjusted model (P for overall = 0.107)(**Figure 2**).

### Machine learning model performance

During the feature selection phase of our machine learning pipeline, eight composite indices were comprehensively evaluated to capture the multidimensional systemic status of the patients. These indices were strategically categorized into three core pathophysiological domains driving autonomic deterioration: (1) systemic immune-inflammatory homeostasis (PLR, NLR, and SII), which reflects the dynamic balance between chronic low-grade inflammation and host immune responses; (2) atherogenic lipid and adiposity burden (AIP, NonHDL_HDL, and eBFP), which quantifies the synergistic effects of lipotoxicity and systemic fat accumulation; and (3) hepatic steatohepatitis and fibrosis risk (FIB4 and BARD score), which captures the detrimental liver-systemic metabolic crosstalk.

**Figure 3:**
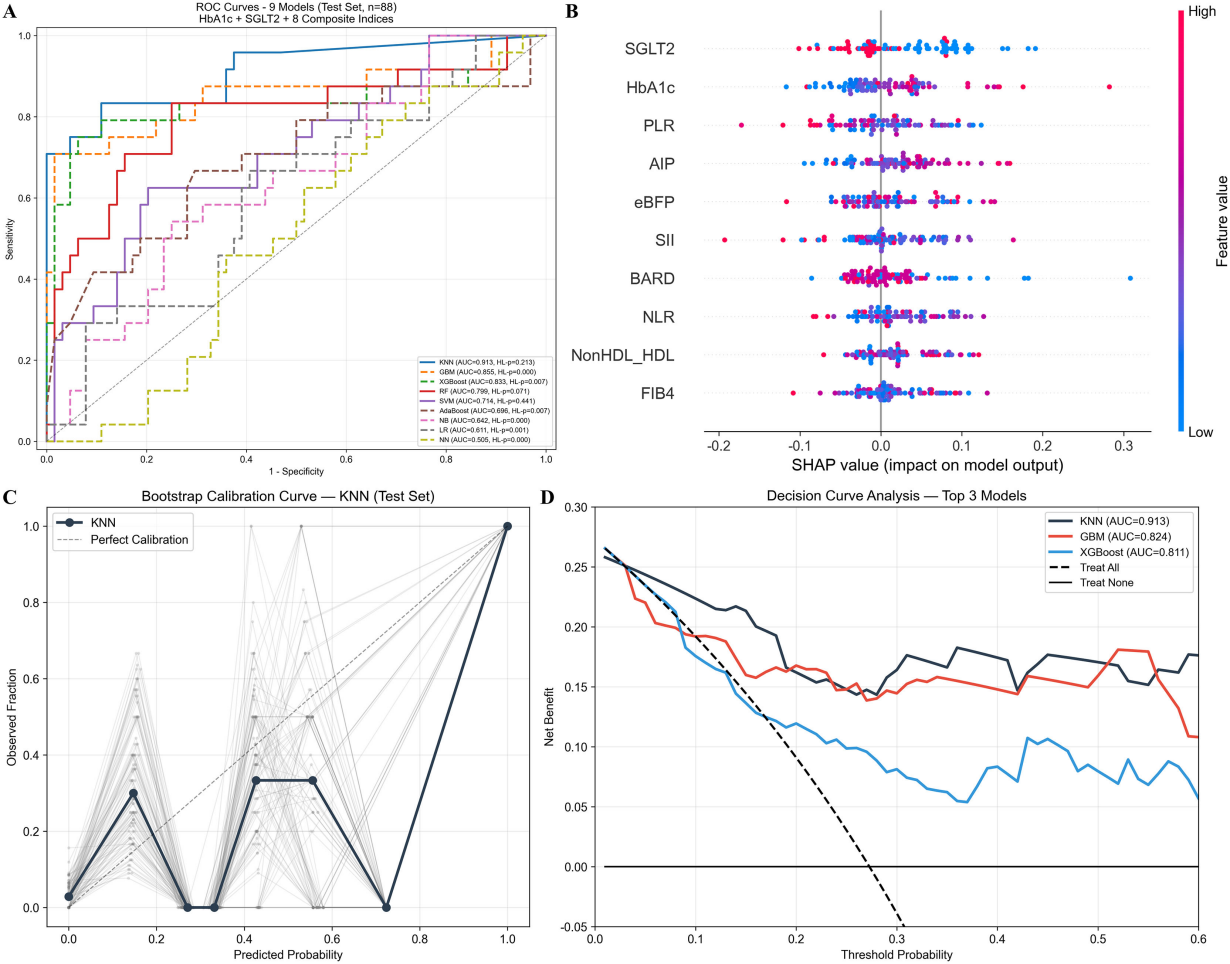
Performance evaluation, feature interpretability, and clinical utility of machine learning models for predicting DCAN deterioration. **(A)** Receiver operating characteristic (ROC) curves comparing the discriminative performance of nine machine learning algorithms on the independent test set. **(B)** SHAP (SHapley Additive exPlanations) summary plot illustrating feature importance and their directional impact on the model output. Each dot represents a single patient, with colors indicating the original feature values (red for high, blue for low). **(C)** Bootstrap calibration curve for the optimal KNN model. **(D)** Decision curve analysis (DCA) of the top three performing models.

Among nine algorithms evaluated with ten features (HbA1c, SGLT2 use, and eight composite indices), K-Nearest Neighbors achieved the highest test-set AUC (0.913), followed by Gradient Boosting Machine (0.855), XGBoost (0.833), Random Forest (0.800), Support Vector Machine (0.714), AdaBoost (0.696), Naive Bayes (0.642), Logistic Regression (0.611), and Neural Network (0.505)(**Figure 3A**).

Calibration assessment, however, revealed substantial discordance between discriminative performance and probability reliability. The Hosmer-Lemeshow test identified significant miscalibration in six of nine models: GBM (P < 0.001), XGBoost (P = 0.007), AdaBoost (P = 0.007), Neural Network (P < 0.001), Naive Bayes (P < 0.001), and Logistic Regression (P < 0.001). Only three models satisfied the pre-specified calibration criterion (H-L P > 0.05): KNN (P = 0.213), SVM (P = 0.441), and RF (P = 0.071). Among these, KNN achieved the highest AUC (0.913) and was therefore selected as the primary predictive model. The KNN model correctly identified 18 of 24 deteriorated patients (sensitivity 75.0%) and correctly excluded 61 of 64 non-deteriorated patients (specificity 95.3%), yielding a positive predictive value of 85.7% (18/21) and a negative predictive value of 91.0% (61/67). Calibration assessment demonstrated acceptable agreement between predicted and observed probabilities (Brier score = 0.084; calibration slope = 0.701; calibration intercept = −0.053) (**Supplementary Table 1**).

Bootstrap internal validation yielded an optimism-corrected AUC of 0.821 (95% CI: 0.704–0.921) for the KNN model, compared with an apparent test-set AUC of 0.913 (optimism = 0.179). DeLong asymptotic testing confirmed that KNN significantly outperformed SVM (P = 0.003), AdaBoost (P < 0.001), Neural Network (P < 0.001), Naive Bayes (P < 0.001), and Logistic Regression (P < 0.001). The AUC advantage of KNN over GBM did not reach statistical significance (Z = 1.421, P = 0.155); however, GBM was excluded by the H-L calibration criterion. Decision curve analysis demonstrated net clinical benefit for KNN across a threshold probability range of 5–40% (**Figures 3C, D**).

SHAP analysis of the KNN model on the test set ranked SGLT2 inhibitor use as the most influential predictor (mean |SHAP| = 0.054), followed by baseline HbA1c (0.045) and PLR (0.044). AIP (0.043), eBFP (0.038), and SII (0.038) showed moderate contributions; NLR (0.034), NonHDL_HDL (0.033), BARD (0.036), and FIB4 (0.031) ranked lowest (**Figure 3B**).

As a robustness check, SHAP analysis using the best-calibrated tree-based model (GBM, TreeExplainer) yielded exact SHAP values: NLR (0.573), HbA1c (0.538), FIB4 (0.515), SII (0.469), AIP (0.435), eBFP (0.354), PLR (0.336), NonHDL_HDL (0.219), SGLT2 (0.098), and BARD (0.058) (**Supplementary Table 2**). HbA1c ranked among the top two features in both KNN and GBM architectures, corroborating the logistic regression findings. BARD consistently ranked at or near the bottom across both models. PLR and AIP demonstrated cross-model robustness as the highest-ranked composite indices.

### Causal mediation analysis between HbA1c and DCAN deterioration

**Figure 4:**
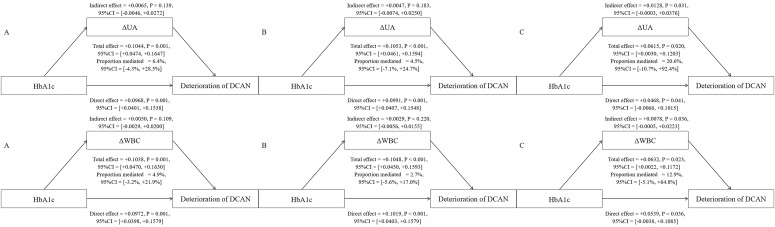
Causal mediation analysis models illustrating the mediating roles of longitudinal changes in uric acid (ΔUA) and white blood cell count (ΔWBC) in the association between HbA1c and DCAN deterioration. **(A–C)** represent the unadjusted crude model (adjusted for nothing) (model 1), the partially adjusted model (adjusted for age, T2DM duration, drinking history and follow up time) (model 2), and the fully adjusted model (adjusted for age, T2DM duration, drinking history, follow up time, hypertension history,SGLT2I use, Baseline Ewing score and Baseline UA/WBC) (model 3), respectively.

Among 36 longitudinal biomarkers systematically evaluated in the fully adjusted model, only follow-up changes in uric acid (ΔUA = UA(follow up) − UA(baseline)) and white blood cell count (ΔWBC = WBC(follow up) − WBC(baseline)) demonstrated nominally significant indirect effects (P < 0.05). The remaining 34 biomarkers showed no significant mediation (all P > 0.05; **Supplementary Table 3**). Causal mediation analysis with 1,000 bootstrap resamples was therefore focused on ΔUA and ΔWBC under three nested adjustment models. In the fully adjusted model, the indirect effect through ΔUA was +0.013 (P = 0.031), accounting for 20.6% of HbA1c’s total effect on DCAN deterioration. The indirect effect through ΔWBC was +0.008 (P = 0.036), accounting for 12.9% of the total effect (**Figure 4**).

## Discussion

Our findings indicate that a high baseline level of HbA1c serves as an independent risk factor for CAN progression. Moreover, mediation analysis elucidated that its adverse effects are partially mediated by dynamic alterations in uric acid and circulating white blood cells counts. However, the estimated indirect associations should not be interpreted as demonstrating that changes in uric acid or white blood cells causally mediate the association between baseline HbA1c and change in the autonomic score. The proposed mediator and outcome changes were measured over the same interval, precluding confirmation that the mediator preceded the outcome. Residual and time-varying confounding, measurement error, and model misspecification also remain possible. As a time-honored and authoritative metric, glycated hemoglobin (HbA1c) reflects the average level of blood glucose fluctuations over a 2 to 3 month period, rather than acting as a mere one-time snapshot like fasting blood glucose (26, 27). Extensive previous research has clearly shown that both glycemic variability and sustained hyperglycemia lead to increased inflammatory cell infiltration and exacerbate the expression of local inflammatory factors. This conclusion has been confirmed not only in cardiovascular diseases but also in many inflammatory diseases to which populations with type 2 diabetes are susceptible (28, 29). Moreover, a secondary analysis of two randomized controlled trials (HALF-PINT and IIT-SBPP) found that the degree of inflammation determines the benefits of glycemic control. Patients in a “hyperinflamed state” had significantly lower mortality after receiving strict glycemic control. Conversely, patients in a low-inflammation state did not benefit from strict glucose lowering (30–32). This perfectly aligns with our finding that changes in white blood cell levels mediate the effect of HbA1c levels on the deterioration of DCAN. High blood glucose and systemic inflammation are not isolated events, but a mutually reinforcing pathological network. Disorders of glucose metabolism exacerbate the inflammatory response, and a high inflammatory state inversely aggravates the injury to target organs (the cardiac autonomic nervous system).

The interplay between glucose dysmetabolism and uric acid homeostasis constitutes a highly complex and bidirectional pathological network. Previous Mendelian randomization evidence indicates that a genetic predisposition to type 2 diabetes and elevated fasting insulin leads to increased serum uric acid levels. Conversely, hyperuricemia acts as an active participant in exacerbating glycemic dysregulation (33). In prediabetic populations, elevated serum uric acid levels not only directly increase fasting plasma glucose but also indirectly elevate HbA1c levels via obesity-related pathways. Notably, even in the presence of normal glucose tolerance, patients with hyperuricemia already exhibit pronounced glycemic fluctuations and heightened insulin resistance, which can be significantly mitigated by urate-lowering therapies (34, 35). Interestingly, as T2DM progresses, despite the persistence of dyslipidemia, the uricosuric effect induced by sustained severe hyperglycemia may result in a paradoxical inverse correlation between fasting glucose and serum uric acid (36). To summary, these findings robustly support our observation: in the evolution of microvascular complications, uric acid is by no means a passive bystander, but rather a crucial mediator bridging glycemic burden and systemic inflammatory cascades.

In this study, rather than pre-selecting potential inflammatory markers, we employed a data-driven machine learning approach to agnostically evaluate eight established composite indices. Notably, the SHAP analysis objectively identified the Platelet-to-Lymphocyte Ratio (PLR) as the most influential composite index for predicting CAN progression, ranking seamlessly behind SGLT2 inhibitor use and HbA1c at baseline. This data-driven identification aligns perfectly with pathophysiological mechanisms. Furthermore, our restricted cubic spline (RCS) analysis revealed a fascinating inverted U-shaped non-linear relationship for PLR, suggesting a threshold effect where initial immune-thrombotic activation exacerbates nerve injury, whereas extreme PLR levels might reflect immune exhaustion or indicate competing risks in end-stage patients, while it is noteworthy that in the fully adjusted RCS model, the overall association of PLR with CAN progression did not reach statistical significance (P = 0.107), and its non-linear trend merely achieved marginal significance (P = 0.050). A previous study utilizing discovery and validation cohorts of patients with type 1 diabetes demonstrated a significant inverse correlation between the immune-mediated glycoprotein soluble urokinase plasminogen activator receptor (suPAR) and objective measures of CAN. Furthermore, chronic low-grade inflammation driven by immune pathways plays a crucial role in the development and progression of CAN (37, 38). Historically, studies investigating the relationship between composite inflammatory indices and the progression of CAN are exceedingly rare. A recent 2024 cross-sectional study examining the correlation of indices such as NLR and PLR with CAN found that although PLR was higher in the CAN group, the difference lacked statistical significance (39). Furthermore, a 2017 study revealed that the relationship between altered inflammatory marker levels and the functional deterioration of CAN was exclusively validated in patients with type 2 diabetes (40).

Several limitations of this study should be acknowledged. First, this was a single-center prospective cohort study, which may potentially limit the generalizability of our findings to other geographic regions or diverse ethnic populations, the findings should therefore not be extrapolated to other populations or healthcare systems without independent external validation and, where necessary, recalibration. Multicenter external validation is a priority for future research. Second, The modest sample size (293 participants and 80 score-increase outcomes), together with comparison of nine algorithms using ten features, creates a risk of model-selection optimism and unstable performance estimates. The 88-participant held-out subset provides internal evaluation only and cannot establish transportability. These results should be considered preliminary until independently reproduced in a larger cohort. Third, despite rigorous stepwise adjustments for key clinical covariates and baseline biomarker levels in our causal mediation models, residual confounding from unmeasured variables, such as detailed dietary habits, physical activity, or other concomitant therapeutic adjustments during follow-up, cannot be entirely ruled out. Fourth, The inverse association between baseline SGLT2 inhibitor use and the worsening outcome should not be interpreted as a treatment effect. Treatment allocation was not randomized, and confounding by indication or contraindication, differences in concomitant care, healthy-user effects, socioeconomic and adherence-related factors, and changes in treatment during follow-up may remain after multivariable adjustment. Residual confounding cannot be excluded. Fifth, The lower baseline Ewing scores in the worsening group requires cautious interpretation. Participants with lower baseline scores have more opportunity for subsequent increases, whereas those with higher scores are constrained by the upper bound of the scale. Regression to the mean and mathematical coupling between baseline score and change may therefore contribute to the observed inverse association. Finally, we did not apply strict multiple testing corrections such as Bonferroni or False Discovery Rate for the 36 longitudinal biomarkers evaluated to avoid obscuring potential mechanistic signals, which inherently increases the risk of Type I errors.

In conclusion, our study demonstrates that a higher baseline glycemic burden is significantly associated with the long-term deterioration of DCAN. Crucially, this association may be partially accounted for by parallel, dynamic pathways involving the longitudinal worsening of uric acid metabolism and systemic inflammation, extending beyond purely direct glucotoxicity. Concurrently, the use of SGLT2 inhibitors is associated with a significantly lower risk of disease progression. Finally, the newly developed and highly calibrated KNN-based machine learning model serves as a promising proof-of-concept tool for early risk stratification, which, pending rigorous external validation, could potentially facilitate personalized, targeted therapeutic interventions to mitigate DCAN progression in high-risk patients.

## Data Availability

The original contributions presented in the study are included in the article/**Supplementary Material**. Further inquiries can be directed to the corresponding authors.
