# Supplementary material for: SGLT2 inhibition, uric acid and inflammation in glycemic burden-related progression of cardiac autonomic neuropathy in type 2 diabetes mellitus: insights from machine learning and mediation analysis
Source: Front Endocrinol (Lausanne). 2026 Aug 5;17:1901683. doi: 10.3389/fendo.2026.1901683 (PMC13485535; doi:10.3389/fendo.2026.1901683)
Supplement: Supplementary file 1 [file Table4.docx]

**STROBE Checklist**

| **No.** | **STROBE item** | **Recommendation** | **Where addressed in the revised manuscript** |
| --- | --- | --- | --- |
| 1 | Title and abstract | (a) State the study design using a standard term in the title or abstract. (b) Give an informative and balanced summary. | Title 1; Abstract 12–15. Partial: add “prospective cohort study” to the title or retain it explicitly; retain cautious calibration wording despite the reported slope 0.701 and intercept −0.053. |
| 2 | Background/rationale | Explain the scientific background and rationale. | Introduction 18–22. |
| 3 | Objectives | State specific objectives and prespecified hypotheses, if applicable. | Abstract—Objective 12; Introduction 22. |
| 4 | Study design | Present key elements of study design early in the paper. | Abstract—Methods 13; Materials and methods 24–26. |
| 5 | Setting | Describe setting, locations, and relevant dates, including recruitment, exposure, follow-up, and data collection. | Methods 25–26; Results 64. Partial: add exact follow-up schedule and median (IQR) plus range in months; current text gives only median 20.000 months. |
| 6 | Participants | Give eligibility criteria, sources and methods of selection, and methods of follow-up. Give matching criteria if applicable. | Methods 27–45; Figure 1 47. Partial: itemize the 222 exclusions and describe selection/follow-up handling; matching not used. |
| 7 | Variables | Clearly define outcomes, exposures, predictors, confounders, effect modifiers, and diagnostic criteria. | Methods 27–43, 50–61. Partial: add SGLT2 agent, dose, duration, adherence, and treatment changes; define timing of follow-up and biomarker changes. |
| 8 | Data sources/measurement | Give data sources and assessment methods for each variable; describe comparability of methods when relevant. | Methods 45, 50–61. Partial: clarify assay/record sources, timing, assessor standardization, and ascertainment of SGLT2 exposure/adherence. |
| 9 | Bias | Describe efforts to address potential sources of bias. | Methods 57–60; Discussion 97. Partial: explicitly address selection/attrition, measurement, confounding by indication, regression to the mean, model optimism, and multiplicity. |
| 10 | Study size | Explain how the study size was arrived at. | Methods 44, 58; Results 64. Partial: state whether this was an available/consecutive cohort and whether no formal sample-size calculation was performed. |
| 11 | Quantitative variables | Explain how quantitative variables were handled, including categorization and cut points where applicable. | Methods 55–57, 61; Table 1. Partial: state explicitly which variables remained continuous, any cut points (or none), and follow-up-time units. |
| 12 | Statistical methods | Describe statistical methods, confounding control, subgroup/interaction analyses, missing-data handling, loss to follow-up, and sensitivity analyses. | Methods 54–61; Results 80–89. Partial: state whether subgroup/interaction analyses were not performed; describe attrition and sensitivity analyses; state multiplicity handling for 36 biomarkers. |
| 13 | Participants—numbers | Report numbers at each study stage and reasons for non-participation; consider a flow diagram. | Results 44; Figure 1 47. Partial: show each exclusion reason and numbers completing each assessment/analysis. |
| 14 | Descriptive data | Give participant characteristics, exposures, confounders, missingness, and follow-up time. | Table 1; Results 44, 64. Partial: add follow-up duration to Table 1 as months (median [IQR]) and range. |
| 15 | Outcome data | Report numbers of outcome events or summary measures over time. | Outcome definition 50; Results 64, 69; Tables 1–2. |
| 16 | Main results | Give unadjusted/adjusted estimates with precision, adjustment variables and rationale, category boundaries, and absolute risk where appropriate. | Table 2; Results 69, 74, 80–84. Partial: prominently report calibration metrics and CIs, give mediation CIs, state reference categories, and reconcile figure/results values. |
| 17 | Other analyses | Report other analyses, including subgroup, interaction, sensitivity, and missing-data analyses. | RCS 56, 74–76; ML/SHAP/validation/DCA 57–60, 78–86; mediation 61, 88–91. Partial: label exploratory analyses and explain multiplicity. |
| 18 | Key results | Summarize key results with reference to study objectives. | Abstract 14–15; Discussion/conclusion 94–98. Partial: revise “highly calibrated” in the conclusion to a cautious description of internal performance. |
| 19 | Limitations | Discuss limitations, potential bias, imprecision, and their direction/magnitude. | Discussion 97. Add loss-to-follow-up/selection bias and variable follow-up if applicable. |
| 20 | Interpretation | Give a cautious interpretation considering objectives, limitations, multiplicity, and relevant evidence. | Discussion 94–98; Abstract 15. Partial: avoid causal SGLT2/mediation language; interpret 20.6% and 12.9% as uncertain, hypothesis-generating mediated proportions. |
| 21 | Generalisability | Discuss external validity/generalisability. | Abstract 15; Discussion 97–98. |
| 22 | Funding | Give funding sources and the role of funders. | Funding 99–100. Partial: add whether funders had any role in design, data, analysis, interpretation, writing, or submission. |
